# Calcium-Alkali Syndrome: Historical Review, Pathophysiology and Post-Modern Update

**DOI:** 10.7759/cureus.13291

**Published:** 2021-02-11

**Authors:** Randa F Zayed, Paul W Millhouse, Farnaz Kamyab, Juan Fernando Ortiz, Adam Atoot

**Affiliations:** 1 Internal Medicine, Hackensack Meridian Health Palisades Medical Center, North Bergen, USA; 2 General Practice, Drexel University College of Medicine, Philadelphia, USA; 3 Architecture, Arts and Humanities, Clemson University, Clemson, USA; 4 Neurology, Universidad San Francisco de Quito, Quito, ECU; 5 Neurology, California Institute of Behavioral Neurosciences & Psychology, Fairfield, USA

**Keywords:** calcium alkali thiazide syndrome, calcium alkali syndrome, milk-alkali, electrolyte disturbances, dietary calcium intake, milk alkali syndrome, electrolyte imbalance, calcium therapy, calcium metabolism, acid-base disorders

## Abstract

Milk-alkali syndrome or calcium-alkali syndrome (CAS) is the triad of hypercalcemia, metabolic alkalosis and renal impairment. It is often related to ingestion of high amounts of calcium carbonate, which was used historically for the treatment of peptic ulcer disease. The incidence of the syndrome decreased dramatically after the introduction of newer peptic ulcer medications such as proton pump inhibitors and histamine blocking agents. However, a resurgence was seen in the late 1980s with the wide use of over-the-counter calcium supplements, mainly by females for osteoporosis prophylaxis. The modern version of the syndrome continues to evolve along with medical management. This review focuses on the historical context of CAS, pathogenesis, resurgence of the condition with variable presentations, and contemporary management.

## Introduction and background

The calcium-alkali syndrome (CAS) consists of hypercalcemia, metabolic alkalosis from the resulting acid/base shift, and various degrees of acute kidney injury. It is typically caused by ingestion of large quantities of calcium with absorbable alkali, such as from taking too many calcium supplements.

The syndrome was first described in reference to patients treated for peptic ulcer disease (PUD) with milk and sodium bicarbonate therapy, hence the term [1]. While this treatment was once a common cause of hypercalcemia, with the introduction of newer peptic ulcer treatment regimens such as histamine-2 blockers and proton pump inhibitors late in the 20th century, the classical milk-alkali syndrome (MAS) nearly disappeared from the medical literature. More recently, however, there has been a resurgence in incidence along with a change in the etiology, largely due to the recent attention to osteoporosis prevention and the availability of over-the-counter calcium carbonate supplements.

Due to the changing etiology and varied presentations, the syndrome can easily be missed. Underdiagnosis has historically been a source of morbidity, potentially leading to permanent renal damage, nephrocalcinosis and metastatic soft-tissue calcifications. This article will discuss the history, pathogenesis, clinical presentation, and management of CAS. There is a focus on the etiology and contemporary causes of this condition and associated pathophysiology.

## Review

Methods and results

Our search began with the U.S. National Library of Medicine website using a Medical Subject Headings (MeSH) query for relevant terms. For milk-alkali syndrome, the MeSH Browser tool suggested “hypercalcemia[MeSH Terms] OR milk alkali syndrome[Text Word]”, whereas no MeSH terms returned for calcium-alkali syndrome. This query was added to the Search Builder and returned 12,995 articles. The PubMed Advanced Search Builder was then used to further refine the results, as shown in Table 1. However, hypercalcemia can occur for a wide variety of reasons, most of which are unrelated to our discussion, and this term was discarded. A combination of the search terms “milk alkali[Text Word]” and “calcium alkali[Text Word]” was found to yield the greatest number of relevant articles.

**Table 1:** PubMed Advanced Search History for relevant terms MeSH, Medical Subject Headings

Search	Query	Filters	Results
#1	"hypercalcemia"[MeSH Terms] OR milk alkali syndrome[Text Word]		12,995
#2	milk alkali[Text Word]		205
#3	calcium alkali[Text Word]		35
#4	#2 OR #3		232
#5	#2 OR #3	English	193
#6	#2 OR #3	English, Humans	152
#7	#2 OR #3	Free full text, Humans, English	40

Filters were then applied and these results were limited to articles written in the English language. If available, the abstracts were reviewed according to the inclusion and exclusion criteria. Only journal articles involving human subjects were included while those discussing microbiological or animal research, or other causes of hypercalcemia or calcinosis, were excluded. The results were also filtered for available free full-text. This resulted in a total of 40 papers.

The following inclusion and exclusion criteria were used in evaluating the papers: (1) articles written in the English language, (2) studies or reports involving human subjects, (3) abstract and full text available, (4) relevance to the subject matter proposed.

Of these results, nine articles were excluded because they did meet the inclusion and exclusion criteria (e.g. did not pertain to the subject matter, was a commentary or editorial), or the information or case material was contained elsewhere. The final number of articles included from this review was 31. For historical perspectives or clarification, where necessary, additional sources were also taken from the bibliographies of these articles. The PubMed search history is shown in Table 1.

Discussion

**Figure 1:**
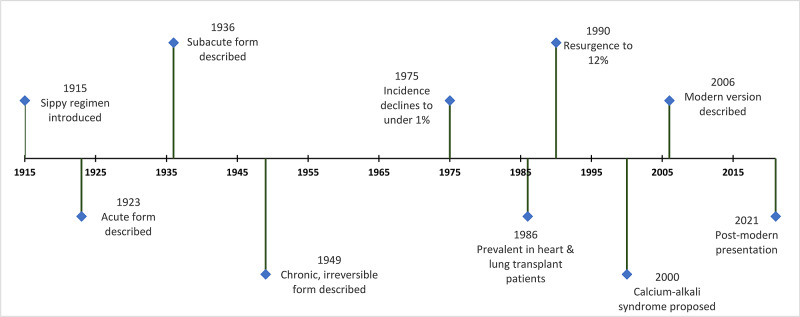
Timeline of important events and publications related to the calcium-alkali syndrome

In regard to the historical overview, for visual context, a timeline of important events and publications is shown in Figure 1.

Early History of the Syndrome

In 1915, a treatment for PUD was introduced by Bertram Sippy [2]. He advocated Schwarz’s dictum of “no acid, no ulcer” [3], and designed a protocol to protect the gastric lining and promote recovery by neutralizing acidic secretions. Sippy’s antacid regimen consisted primarily of milk or cream along with Sippy powders (combinations of magnesium carbonate, sodium bicarbonate, bismuth subcarbonate and sodium bicarbonate), as well as intermittent gastric aspiration. He reported excellent response and improvement of gastric ulcers, and milk-alkali therapy subsequently became the standard of care treatment for PUD [2].

Toxic reactions were soon observed in patients using this regimen [4,5]. In 1923, a review by Hardt and Rivers concluded that patients with duodenal ulcers managed with the Sippy method may develop symptoms of toxemia along with renal changes and elevations in the serum nitrogen and carbon dioxide [4]. In 1936, a report by Cope associated the regimen with a syndrome of hypercalcemia, alkalosis and kidney injury [1]. Cope found that the electrolyte imbalances and azotemia would completely resolve upon discontinuation of therapy [1]. Reports from the 1920s and ’30s further characterized the adverse effects associated with the Sippy regimen, with an incidence ranging from 2% to 18% [6,7] and a mortality rate of 4.4% [5-8].

In a study of 1,350 patients with PUD, 8% developed the characteristic syndrome after treatment with the Sippy protocol [5]. In 1949, Burnett and Commons described six cases of male patients who had been taking large quantities of milk and resorbable alkali for extended periods of time (2-30 years) for peptic ulcers. The reported adverse events included hypercalcemia with normal alkaline phosphatase levels, alkalosis, renal insufficiency, and calcinosis (in the cornea, subcutaneous tissue, lung, falx cerebri and lymph nodes) [5]. In this series, chronic renal failure developed resulting in four deaths.

Later reports continued to investigate and characterize MAS. It was believed that the prior reports likely represented distinct stages of the disease, progressing from mild with complete resolution of symptoms [4], to intermediate [1], and finally to a severe, irreversible form [5]. A review on the basis of chronicity proposed classification of MAS into subtypes: Cope syndrome (reversible, acute form), and Burnett syndrome (irreversible, chronic form), with the latter involving the presence of metastatic soft tissue calcifications [9,10].

Changing Incidence and Etiology

The prevalence of MAS remained high through the 1950s and ’60s, with various review articles written to define disease characteristics [9,11]. However, the incidence of MAS declined following the introduction of nonabsorbable antacids and later histamine-2 blockers. In 1975, Jamieson reported an incidence of MAS in less than 1% of hypercalcemia cases [12]. During this period, MAS had virtually vanished as a clinical entity.

Throughout the 1970s and ’80s, the incidence remained low with reported rates of under 2% of cases of hypercalcemia [12,13]. In these cases, however, the syndrome was more often associated with calcium carbonate administration and occurred in patients with conditions predisposing them to hypercalcemia (e.g., vomiting, hypokalemia, hypertension, renal failure, hemorrhage, or diuretic use). Also, the serum phosphate level was significantly lower than historical reports. The most likely explanation for this shift was that the more recent cases of MAS were related to calcium carbonate supplements rather than the overconsumption of dairy products (which have high phosphate content), formerly, the most common source of dietary calcium [10].

Despite the previous decline, the incidence of MAS dramatically increased in the 1990s. Prior to 1989, MAS was present in just 2% of patients admitted to the hospital, whereas it was found in 12% of inpatients between 1990 and 1993 [13]. Another study from the period 1998 to 2003 found MAS in 8.8% of all hospitalized patients [14].

As the incidence of MAS increased, the etiology also changed, and there was no longer a male predominance. In a retrospective review of transplant patients medicated with steroids and 8-12 g of calcium carbonate daily for osteoporosis prophylaxis, hypercalcemia was present in 20%, and approximately half developed alkalosis [15]. The pooled data from 53 cases of MAS reported from 1983 through 2004 showed that all of them were taking calcium carbonate supplementation and only a minority consumed large quantities of milk. This data further demonstrates the shifting etiology of MAS. Permanent renal insufficiency was observed in over a third of the patients, which suggests diagnostic delays and thus a low index of suspicion [10].

Another cause of MAS found primarily in Asia and the South Pacific is the consumption of betel nuts with a calcium alkali paste [16]. In this population, the prevalence of MAS ranges from 9% to 12% and it is the third most common cause of hypercalcemia after hyperparathyroidism and malignancy [16,17].

Modern Version

In 2006, a “modern version” of MAS was described by Beall et al. [10]. Calcium supplements rather than dairy products were the primary causative factor of MAS, and the modern version involved new clinical parameters and case presentations. The reemergence of the syndrome, while multifactorial in nature, was proposed to involve the increased usage of calcium carbonate for managing dyspepsia, for the prevention of osteoporosis in postmenopausal women and those on long-term corticosteroid therapy, and for chronic renal failure patients. The trending awareness of osteoporosis and increasing availability of over-the-counter supplements containing calcium and non-absorbable alkali also contributed to this phenomenon [10].

The modern version differed from the classical description in various ways. The patients were more often asymptomatic with the finding of hypercalcemia, alkalosis, or renal impairment being incidental. Hypophosphatemia was common, whereas historically the calcium loading in MAS involved milk, which contains a large amount of phosphate. Hypomagnesemia was also common, perhaps related to the effect of hypercalcemia on inhibiting magnesium reabsorption by the renal tubule [14]. Parathyroid hormone (PTH) levels were generally low, which can help differentiate MAS from primary hyperparathyroidism [14].

Post-Modern Presentation

More than a decade has now elapsed since the modern version of MAS was described. Over that time, our search yielded 23 case reports discussing a similar syndrome. The etiology in these cases was predominantly calcium-containing supplements. A list of potential triggers now includes medications that affect kidney perfusion (angiotensin receptor blockers, sartans, aldosterone receptor antagonists, sulfonamide-derived diuretics, anthranoid laxatives [18]), changes in calcium carbonate supplementation, a diet rich in pH-basic foods (i.e. ,vegetarianism), dehydration, pregnancy and the influenza vaccine [19,20]. Other contemporary associations with MAS include hypokalemia, anorexia nervosa, bulimia, primary hyperparathyroidism or hypoparathyroidism (management-induced), pregnancy, PUD, gastroesophageal reflux disease, pre-existing renal disease, Munchausen syndrome, and hydrochlorothiazide therapy [10]. A case was reported in which MAS developed due to nicotine-replacement gum and carbonated water [21]. Another case involved the administration of alfacalcidol and a thiazide diuretic, suggesting that the oral intake of calcium and alkali is not strictly necessary to cause the syndrome [22]. There are also reports of bone resorption following immobilization leading to hypercalcemia and alkalosis [23].

The presentations and pathophysiology of MAS continue to evolve. The demographics now comprise mostly pregnant and post-menopausal women, bulimic patients, solid organ transplant recipients, and those on dialysis [24]. Due to the changing etiopathology, many authors have suggested changing the name to calcium-alkali syndrome as proposed by Sulkin and Krentz in 2000 [24-26]. One group proposed further classification into calcium-alkali-thiazide syndrome given the frequency of this association [27]. Milk-alkali syndrome is an antiquated term referring to a historic treatment for PUD and does not adequately reflect the range of causes and presentations, possibly leading to missed or delayed diagnoses. For these reasons, current perspectives support and emphasize the new terminology. CAS now accounts for more than 10% of cases of hypercalcemia overall, and it is the third most common cause of hypercalcemia among hospitalized patients [26].

Pathophysiology

The primary defining feature of CAS is the ingestion of calcium and absorbable alkali leading to the triad of hypercalcemia, alkalosis, and renal impairment [9]. In healthy individuals, excessive ingestion of calcium affects calcitriol levels that in turn reduces intestinal calcium absorption. If calcium intake increases to as much as 10-15 g per day, calcitriol suppression is insufficient to maintain normal calcium levels. Net calcium absorption eventually leads to hypercalcemia [28-30]. In some cases, a dose of under 5 g calcium carbonate daily led to CAS [11,29,30]. In addition to excess consumption, other pathogenetic processes such as variances in calcium homeostasis and renal tubular reabsorption, abnormalities of vitamin D metabolism, and differences in alkaline secretion also play a role in the development of CAS [10].

Calcium metabolism is the maintenance of appropriate levels of calcium in the body. This involves the intake (via the gut) and output (through the gut and kidneys) of calcium as well as regulation thereof between compartments such as blood plasma, extracellular and intracellular fluid, and bone. The skeletal system acts as a calcium storage bank, holding the vast majority (~98%) of the total body calcium. The remaining ~2% circulates in the plasma either bound to albumin or complexed with citrate, sulfate, or phosphate. Approximately 45% can be found as ionized or free calcium. Calcium homeostasis is the regulation of calcium ions in the blood plasma. This involves a negative feedback mechanism as the biologic effect is determined by the amount of ionized calcium (rather than total). The two hormones implicated are PTH and calcitonin, which are released to raise or lower serum free calcium levels, respectively [31].

Calcium absorption involves both active and passive processes. Passive absorption is a concentration-dependent paracellular process located mainly in the jejunum and ileum that occurs with sufficient calcium intake [28]. Active absorption is a transcellular process located mainly in the duodenum and upper jejunum that is upregulated when calcium intake is low. Active absorption is largely regulated by renal hydroxylation of the mostly inactive, plant-based calcidiol (25-hydroxycholecalciferol, vitamin D2), whereby lower concentrations of the active animal hormone calcitriol (1,25-dihydroxycholecalciferol, vitamin D3) will reduce the fractional calcium absorption [11,31,32].

These regulation and feedback mechanisms work in concert to control the absorption of calcium and should theoretically prevent development of hypercalcemia in normal subjects. However, the modulation of calcium absorption in the presence of high intake varies from patient to patient, and there are so-called hyper-absorbers that generally uptake more fractional calcium than others [9,10]. Calcium absorption by the small intestine is also affected by gastric acidity, age, and the concentration of ingested calcium products [32]. The calcium buffering capacity of bone, which involves osteoblasts and osteoclasts and is hormonally regulated by PTH and calcitriol, also varies between individuals [33].

The kidneys play a role in maintaining calcium homeostasis as well. Ionized calcium is filtered by the glomerulus and reabsorbed in the proximal tubule and thick ascending loop of Henle. In usual physiologic conditions, the renal excretion of calcium generally responds to serum concentrations, and gastric absorption is affected to maintain appropriate levels. However, prolonged hypercalcemia may reduce the renal excretory capacity and disrupt the regulatory system. Hypercalcemia has various effects on the kidney that result in hypovolemia [26]. Elevated serum calcium causes vasoconstriction of the renal afferent arteriole that alters kidney function and impairs the concentrating ability of the renal tubules [34]. Sustained hypercalcemia can reduce glomerular filtration rate, suppress PTH, impair creatinine clearance, and ultimately lead to renal insufficiency.

Contraction alkalosis refers to the increase in blood pH that occurs as a result of reduced volume. In the short term, a reduction in blood volume has little change in bicarbonate. Over the long term, renal compensatory mechanisms are involved - renin, angiotensin II, and aldosterone are all increased. This results in increased sodium-hydrogen ion exchange and bicarbonate reabsorption in the proximal tubule, as well as increased potassium ion secretion in the collecting duct. The effects are chloride depletion and hypokalemia.

Increased bicarbonate reabsorption leads to metabolic alkalosis [24]. Alkalosis in turn increases the hypercalcemia by enhancing calcium reabsorption in the distal nephron [24]. Other effects of hypercalcemia include the activation of calcium-sensing receptors in the parathyroid gland that reduces PTH through negative feedback. This leads to a reduction of calcium reabsorption in the kidneys. Hypercalcemia causes impairment in aquaporin-2 expression leading to the blockage of antidiuretic hormone (ADH)-dependent water reabsorption and diuresis [35]. The resulting sodium excretion leads to further depletion of the total body water. The volume depletion stimulates absorption of bicarbonate in an effort to maintain extracellular volume leading to metabolic alkalosis [36]. Alkalosis then facilitates more hypercalcemia and volume depletion, further reducing renal blood flow and compounding the cycle [37,38].

Paradoxical aciduria is often seen in the final stages of acute CAS [10]. As the volume depletion and metabolic alkalosis continue, there is a complete resorption of sodium and bicarbonate with severe volume depletion despite alkalosis [10]. The effect of hypercalcemia on impairing renal function and suppressing PTH hormone also leads to increased phosphate levels [34]. Suppressed PTH increases renal bicarbonate resorption that contributes to the alkalosis [39]. Together, hypercalcemia and hyperphosphatemia cause damage to renal tubular cells, thickening of basement membrane, cellular necrosis and calcium deposition. If not treated appropriately, nephrocalcinosis and irreversible renal impairment can develop [5].

Clinical Manifestations

The presentation of MAS varies based on the duration and quantity of supplementation and the presence of concomitant factors such as thiazide diuretics or volume depletion. MAS classically presented with symptomatic hypercalcemia, although the symptomatology varied. Recently, CAS has been an incidental finding. Patients are often asymptomatic and found to have hypercalcemia, alkalosis, and renal function impairment during workup for another reason. It is also common to find hypomagnesemia and low to normal phosphate levels [14]. Although CAS is diagnosed clinically, it is important to rule out other causes of hypercalcemia. A high index of suspicion should be held for the syndrome including a detailed review of any over-the-counter medications and a thorough laboratory workup [13].

The classical description of MAS was classified into three clinical forms based on severity and associated clinical entities. The laboratory abnormalities were similar in each although the response to treatment varied:

Acute: The acute or toxemic syndrome could occur after just one week of milk-alkali treatment and presented with symptoms of acute hypercalcemia (such as nausea, vomiting, headache, dizziness, pruritus, weakness, sensory alteration and confusion). In addition, there was often severe metabolic alkalosis, renal impairment, and normal or elevated phosphate levels. Increased urinary calcium or decreased phosphate levels would occasionally be seen. ECG changes included QT/QTc interval shortening and often nonhypothermic J waves [40]. The acute form resolved with withdrawal of milk and alkali therapy [8].

Subacute: The subacute or intermediate form (also called Cope’s syndrome) occurred in patients that took milk-alkali therapy intermittently for extended periods. The patients presented with symptoms of both acute and chronic hypercalcemia. Gradual clinical improvements were seen after withdrawal of therapy. In some cases, renal function would remain mildly impaired [9].

Chronic: The chronic syndrome (also called Burnett’s syndrome) affected patients with long histories of high milk and alkali ingestion. Patients presented with symptoms of chronic hypercalcemia such as polyuria, polydipsia, tremor, psychosis, muscle aches, and pruritus [5,41,42]. There was often evidence of calcinosis of various organs, including nephrocalcinosis and metastatic calcifications of the cornea (band keratopathy), kidneys, and nasal and temporal areas. Patients would have slow resolution of symptoms after therapy cessation. The renal impairment usually persisted and many patients developed chronic kidney disease [5].

The classical laboratory findings in MAS included elevated serum calcium, normal or increased serum phosphate, and metabolic alkalosis [5]. The similarity of these findings with those of hyperparathyroidism has historically been a source of confusion [43]. The method of choice for diagnosing hyperparathyroidism is measurement of intact PTH. The earlier single-antibody immunoassays had poor discrimination [44] or could be elevated in the presence of reduced renal function. Labs now measure PTH via intact two-antibody assays for more accurate results. As expected, PTH is reduced in the presence of hypercalcemia due to physiologic suppression [13]. However, the PTH depression is present only when there is sustained high levels of serum calcium [45]. A resulting rebound can occur with PTH levels increasing within hours of discontinuing exogenous calcium and peaking around one week later before normalizing [10,13]. This fluctuation in PTH levels will affect the serum calcium as well, resulting in an initial reduction to subnormal levels before gradually returning to normal. Hence, an accurate interpretation of PTH levels must also consider the timing of measurement [10].

One might be tempted to use the serum 1,25-(OH)D levels to help differentiate CAS from primary hyperparathyroidism. Hypercalcemia will lower 1,25-(OH)D and intact PTH levels secondary to a negative feedback mechanism [45]. Thus, in CAS, both the vitamin D and PTH levels will be low, whereas in primary hyperparathyroidism, both measurements are usually elevated [46]. However, there are reports of CAS cases in which vitamin D levels were not suppressed [43]. Also, elevated PTH levels have been found in some cases of CAS leading to misdiagnosis of the syndrome as primary hyperparathyroidism. A study by Beal and Scofield found that patients with CAS underwent unnecessary parathyroid testing in 10% of cases [13]. The interpretation of lab values in cases of suspected CAS should be done cautiously and take into account the timing of interventions. Other assays such as the serum calcium, magnesium, phosphate, vitamin D, and intact PTH levels may be considered in the setting of the overall clinical picture [10].

A partial differential diagnosis with the expected lab values in cases of hypercalcemia is shown in Table 2.

**Table 2:** Expected laboratory findings in common causes of hypercalcemia ↑, increased; ↓, decreased; -, normal; ~, variable; Ca, calcium; PTH, parathyroid hormone; PO_4_, phosphate; 25-(OH)D, 25-hydroxyvitamin D; 1,25-(OH)D, 1,25-dihydroxyvitamin D

Disease	Ca	PTH	PO_4_	25-(OH)D	1,25-(OH)D
Hyperparathyroidism (1° and 3°)	↑	↑↑	↓	↓ to -	↑
Familial hypocalciuric hypercalcemia	↑	↑ or -	↓	-	-
Malignancy	↑	↓	~	~	~
Vitamin D excess	↑	↓	↑	↑	~
Milk-alkali syndrome	↑	↓	↓	-	-
Increased bone turnover	↑	↓	↑	~	~

Management

In most cases, supportive therapy with isotonic saline and offending agent withdrawal usually results in the resolution of hypercalcemia and metabolic alkalosis. With significant elevations of calcium, such as when CAS manifests as a hypercalcemic crisis, intravenous furosemide, pamindronate, and hydrocortisone can be used [30,47,48]. For severe hypercalcaemia and metabolic alkalosis in the setting of acute renal insufficiency, urgent dialysis, loop diuretics, and calcitonin may be considered [18]. Calcium levels typically normalize within several days although they can remain elevated for up to six months [43]. A slow recovery of PTH can sometimes lead to temporary symptomatic hypocalcemia (a feature possibly unique to CAS [49,50]) that may require replacement therapy [13]. Renal function generally returns to normal if the diagnosis of CAS is made early in the disease course. In patients with irreversible renal insufficiency, hemodialysis may be required.

## Conclusions

Milk-alkali syndrome, now referred to as calcium-alkali syndrome, is an interesting clinical entity that continues to evolve over time. A common ailment in the 20th century due to the popular “Sippy” regimen, the incidence of the syndrome decreased dramatically after the introduction of newer peptic ulcer treatments. However, there has been a reemergence recently, primarily due to the use of over-the-counter supplements. Today the syndrome is common in post-menopausal women and others taking calcium carbonate, mainly for osteoporosis prevention. The clinical presentation also differs from the classical description as patients are more likely to be asymptomatic. The syndrome can even be found in the absence of ingestion of either calcium or an alkali source. The differential diagnosis includes hypoparathyroidism, malignancy, and other disorders involving calcium metabolism. Laboratory analyses such as serum phosphate and vitamin D levels and intact dual-antibody PTH assays may help make the distinction. The interpretation of lab values in cases of suspected CAS should always involve the timing of interventions as signs and symptoms can vary temporally and with treatments.

## References

[1] Cope C (1936). Base changes in the alkalosis produced by the treatment of gastric ulcer with alkalies. Clin Sci.

[2] Sippy BW (1915). Gastric and duodenal ulcer: medical cure by an efficient removal of gastric juice corrosion. JAMA.

[3] Isenberg JI, Spector H, Hootkin LA, Pitcher JL (1971). An apparent exception to Schwarz's dictum, "no acid--no ulcer". N Engl J Med.

[4] Hardt LL, Rivers AB (1923). Toxic manifestations following the alkaline treatment of peptic ulcer. Arch Intern Med.

[5] Burnett CH, Commons RR (1949). Hypercalcemia without hypercalcuria or hypophosphatemia, calcinosis and renal insufficiency; a syndrome following prolonged intake of milk and alkali. N Engl J Med.

[6] Rafsky HA, Schwartz L, Kruger AW (1932). The relation of alkalosis to peptic ulcer. JAMA.

[7] Gatewood WE, Gaebler OH, Muntwyler E, Myers VC (1928). Alkalosis in patients with peptic ulcer. Arch Intern Med.

[8] Cooke AM (1932). Alkalosis occurring in the alkaline treatment of peptic ulcers. Q J Med.

[9] Punsar S, Somer T (1963). The milk-alkali syndrome. A report of three illustrative cases and a review of the literature. Acta Med Scand.

[10] Beall DP, Henslee HB, Webb HR, Scofield RH (2006). Milk-alkali syndrome: a historical review and description of the modern version of the syndrome. Am J Med Sci.

[11] Wenger J, Kirsner JB, Palmer WL (1957). The milk-alkali syndrome: hypercalcemia, alkalosis and azotemia following calcium carbonate and milk therapy of peptic ulcer. Gastroenterology.

[12] Jamieson M (1985). Hypercalcaemia. Br Med J (Clin Res Ed).

[13] Beall DP, Scofield RH (1995). Milk-alkali syndrome associated with calcium carbonate consumption. Report of 7 patients with parathyroid hormone levels and an estimate of prevalence among patients hospitalized with hypercalcemia. Medicine (Baltimore).

[14] Picolos MK, Lavis VR, Orlander PR (2005). Milk-alkali syndrome is a major cause of hypercalcaemia among non-end-stage renal disease (non-ESRD) inpatients. Clin Endocrinol (Oxf).

[15] Kapsner P, Langsdorf L, Marcus R, Kraemer FB, Hoffman AR (1986). Milk-alkali syndrome in patients treated with calcium carbonate after cardiac transplantation. Arch Intern Med.

[16] Wu KD, Chuang RB, Wu FL, Hsu WA, Jan IS, Tsai KS (1996). The milk-alkali syndrome caused by betelnuts in oyster shell paste. J Toxicol Clin Toxicol.

[17] Allen SE, Singh S, Robertson WG (2006). The increased risk of urinary stone disease in betel quid chewers. Urol Res.

[18] Morini L, Donelli D, Santi R, Trenti C, Battaglino G, Iannuzzella F, Negri EA (2017). Severe milk-alkali syndrome in a patient with hypoparathyroidism associated with 1,25(OH)2D, hydrochlorothiazide and anthranoid laxative consumption. Eur J Case Rep Intern Med.

[19] Skwarek A, Pachucki J, Bednarczuk T, Żurecka Z, Popow M, Kondracka A, Bartoszewicz Z (2018). Milk-alkali syndrome (MAS) as a complication of the treatment of hypoparathyroidism - a case study. Endokrynol Pol.

[20] Al-Hwiesh AK, Abdul-Rahman IS, Al-Oudah N, Al-Solami S, Al-Muhanna FA (2017). Milk-alkali syndrome induced by H1N1 influenza vaccine. Saudi J Kidney Dis Transpl.

[21] Swanson CM, Mackey PA, Westphal SA, Argueta R (2013). Nicotine-substitute gum-induced milk alkali syndrome: a look at unexpected sources of calcium. Endocr Pract.

[22] Satoh F, Okado T, Iwamoto M (2010). Calcium-alkali syndrome-like symptoms manifested by daily alphacalcidol and thiazide. Intern Med.

[23] Kleinig TJ, Torpy DJ (2004). Milk-alkali syndrome: broadening the spectrum of causes to allow early recognition. Intern Med J.

[24] Stojceva-Taneva O, Taneva B, Selim G (2016). Hypercalcemia as a cause of kidney failure: case report. Open Access Maced J Med Sci.

[25] Sulkin T, Krentz AJ (2000). Iatrogenic recurrent severe hypercalcaemia and renal impairment. Postgrad Med J.

[26] Ali R, Patel C (2020). Milk-alkali syndrome. In StatPearls.

[27] Rehan MA, Rashid A, Krell K, Gabutti C, Singh R (2020). Calcium alkali thiazide syndrome: what we need to know. Cureus.

[28] Bronner F (2003). Mechanisms of intestinal calcium absorption. J Cell Biochem.

[29] Orwoll ES (1982). The milk-alkali syndrome: current concepts. Ann Intern Med.

[30] Newmark K, Nugent P (1993). Milk-alkali syndrome: a consequence of chronic antacid abuse. Postgrad Med.

[31] Peacock M (2010). Calcium metabolism in health and disease. Clin J Am Soc Nephrol.

[32] Adams ND, Gray R, Lemann J Jr (1979). The effects of oral CaCO3 loading and dietary calcium deprivation on plasma 1, 25-dihydroxyvitamin D concentrations in healthy adults. J Clin Endocrinol Metab.

[33] Felsenfeld AJ, Levine BS (2006). Milk alkali syndrome and the dynamics of calcium homeostasis. Clin J Am Soc Nephrol.

[34] Lemann J Jr, Adams ND, Gray RW (1979). Urinary calcium excretion in human beings. N Engl J Med.

[35] Khositseth S, Charngkaew K, Boonkrai C (2017). Hypercalcemia induces targeted autophagic degradation of aquaporin-2 at the onset of nephrogenic diabetes insipidus. Kidney Int.

[36] Brown EM (1999). Physiology and pathophysiology of the extracellular calcium-sensing receptor. Am J Med.

[37] Sutton RA, Wong NL, Dirks JH (1979). Effects of metabolic acidosis and alkalosis on sodium and calcium transport in the dog kidney. Kidney Int.

[38] Patel AM, Goldfarb S (2010). Got calcium? Welcome to the calcium-alkali syndrome. J Am Soc Nephrol.

[39] Morris RC, Sebastian A, McSherry E (1972). Renal acidosis. Kidney Int.

[40] Chhabra L, Spodick DH (2013). Milk alkali syndrome: an electrocardiographic masquerader for non-hypothermic Osborn phenomenon. Heart.

[41] Junor BJ, Catto GR (1976). Renal biopsy in the milk-alkali syndrome. J Clin Pathol.

[42] Kallmeyer JC, Funston MR (1983). The milk-alkali syndrome: a case report. S Afr Med J.

[43] Carroll PR, Clark OH (1983). Milk alkali syndrome. Does it exist and can it be differentiated from primary hyperparathyroidism?. Ann Surg.

[44] Arnaud CD, Goldsmith RS, Bordier PJ, Sizemore GW (1974). Influence of immunoheterogeneity of circulating parathyroid hormone on results of radioimmunoassays of serum in man. Am J Med.

[45] Medarov BI (2009). Milk-alkali syndrome. Mayo Clin Proc.

[46] Abreo K, Adlakha A, Kilpatrick S, Flanagan R, Webb R, Shakamuri S (1993). The milk-alkali syndrome. A reversible form of acute renal failure. Arch Intern Med.

[47] Vanpee D, Delgrange E, Gillet JB, Donckier J (2000). Ingestion of antacid tablets (Rennie®) and acute confusion. J Emerg Med.

[48] Tal A, Powers K (1996). Milk-alkali syndrome induced by 1,25(OH)2D in a patient with hypoparathyroidism. J Natl Med Assoc.

[49] Fiorino AS (1996). Hypercalcemia and alkalosis due to the milk-alkali syndrome: a case report and review. Yale J Biol Med.

[50] Morton A (2002). Milk-alkali syndrome in pregnancy, associated with elevated levels of parathyroid hormone-related protein. Intern Med J.

